# T Cell Responses to BA.2.86 and JN.1 SARS-CoV-2 Variants in Elderly Subjects

**DOI:** 10.3390/vaccines12121451

**Published:** 2024-12-23

**Authors:** Irene Segato, Dalila Mele, Greta Forlani, Daniela Dalla Gasperina, Mario U. Mondelli, Stefania Varchetta

**Affiliations:** 1 PhD National Programme in One Health Approaches to Infectious Diseases and Life Science Research, Department of Public Health, Experimental and Forensic Medicine, University of Pavia, 27100 Pavia, Italy; irene.segato01@universitadipavia.it; 2 Division of Microbiology and Virology, Department of Diagnostic Services, Fondazione IRCCS Policlinico San Matteo, 27100 Pavia, Italy; d.mele@smatteo.pv.it; 3 Laboratory of General Pathology and Immunology “Giovanna Tosi”, Department of Medicine and Technological Innovation, University of Insubria, 21100 Varese, Italy; greta.forlani@uninsubria.it; 4 Department of Medicine and Technological Innovation ASST Sette Laghi, University of Insubria, 21100 Varese, Italy; d.dallagasperina@uninsubria.it; 5 Department of Internal Medicine and Therapeutics, University of Pavia, 27100 Pavia, Italy; 6 Division of Clinical Immunology—Infectious Diseases, Fondazione IRCCS Policlinico San Matteo, 27100 Pavia, Italy; 7 Infectious Diseases Unit, Department of Internal Medicine, Fondazione IRCCS Ca’ Granda Ospedale Maggiore Policlinico, 20122 Milano, Italy

**Keywords:** SARS-CoV-2, COVID-19, vaccine, BA.2.86, JN.1, elderly

## Abstract

Background/Objectives: New SARS-CoV-2 variants are continuously emerging, making it essential to assess the efficacy of vaccine-induced immune protection. Limited information is available regarding T cell responses to BA.2.86 and JN.1 variants, particularly in elderly individuals. Methods: We evaluated T cell and total IgG responses against the receptor-binding domain (RBD) of the ancestral SARS-CoV-2 strain, as well as BA.2.86 and JN.1 omicron subvariants, in two groups of subjects. One group consisted of SARS-CoV-2-exposed elderly individuals who were fully vaccinated with the BNT162B2 mRNA vaccine, with a booster dose of the updated 2023–2024 COVID-19 vaccine (XBB.1.5) at least 15 days after receiving a booster dose of the updated 2023–2024 COVID-19 vaccine. The second group consisted of healthcare workers who were unexposed to SARS-CoV-2 one month after the booster dose of the first-generation BNT162b2 mRNA vaccine. T cell activation-induced markers (AIM) and IFN-γ secretion were evaluated by flow cytometry and ELISpot assays, respectively. Results: Elderly subjects showed reduced IgG levels against JN.1 compared with the ancestral strain. BA.2.86 stimulation resulted in lower IFN-γ levels in the elderly versus the COVID-19-naïve group. AIM analysis showed that among T cells, CD4+ were the most responsive, with a reduced proportion of JN.1-reactive CD4+ T cells compared with the ancestral strain in the SARS-CoV-2-unexposed group. Despite receiving the updated booster, the elderly group showed reduced CD4+ T cell reactivity to BA.2.86. Conclusions: The XBB.1.5-containing vaccine induced lower CD4+ T cell responses against BA.2.86 in the elderly. CD4+ T cells from BNT16b2-vaccinated, COVID-19-naïve subjects recognized ancestral and BA.2.86 RBD strains while showing reduced responses to JN.1. These results emphasize the need for tailored vaccine strategies for emerging variants, particularly in vulnerable populations.

## 1. Introduction

mRNA vaccines have revolutionized the course of SARS-CoV-2 infection, saving millions of lives. The first vaccines were developed less than one year after the onset of the first pandemic wave, demonstrating an unprecedented speed of development. The virus seems to have hit a dead end with the omicron variant, acquiring less aggressive features and by and large limiting the extension to the upper respiratory tract. However, omicron subvariants are continuously emerging, posing challenges to vaccine efficacy. Indeed, different studies have shown that primary vaccinations and initial boosters exhibit reduced efficacy against Omicron and its subvariants [1,2,3]. The updated 2023 vaccine targeting the XBB.1.5 variant also showed decreased efficacy against newer variants that emerged later in 2023, including EG.5, XBB.1.16, BA.2.86, and JN.1 [4,5]. This highlights the ongoing struggle between the emergence of new variants and the effectiveness of vaccines and further emphasizes the concept that COVID-19 vaccines are unable to provide sterilizing immunity. Mutations may affect the virus’s ability to infect cells, to propagate the infection, and to evade the host’s immune responses. Indeed, it has been shown that Omicron variants increase innate immune evasion by increasing viral protein expression [6,7]. Several SARS-CoV-2 variants caused significant outbreaks of infection since the emergence of the ancestral wild-type strain, SARS-CoV-2, in late 2019 (Supplementary Figure S1). In August 2023, the BA.2.86 subvariant emerged in Denmark and Israel and rapidly spread in several countries [8]. Compared to the XBB.1.5 omicron subvariant, BA.2.86 carries 35 mutations within the spike protein [8], including 10 within the receptor-binding domain (RBD), and shows increased receptor affinity and enhanced lung cell infectivity [9,10]. Despite these advantages, BA.2.86 did not become prevalent, possibly due to its relatively lower immune evasion compared to the XBB.1.5 strain [11]. During the following months, the BA.2.86 descendant JN.1 (BA.2.86.1.1), characterized by a solitary additional mutation in the S protein, L455S, emerged as the predominant lineage [12]. L455S is located in the RBD, in a key place for viral binding to its ligand, ACE2. Surprisingly, although JN.1 showed increased infectivity, its binding affinity for ACE2 was reduced compared to BA.2.86 [13,14].

However, some reports indicated that, despite its reduced capacity to bind the receptor, the L455S mutation conferred increased resistance to neutralizing antibodies (NAbs), enhancing the virus’s ability to evade immune responses [13,14].

Given the continuous emergence of viral variants, vaccines have been updated over time to target new variants. The last vaccine, targeting the XBB.1.5 omicron subvariant, was introduced in September 2023. However, COVID-19 vaccine coverage in 2023–2024 was notably lower than during the initial campaign. The recent ECDC report on COVID-19 vaccine coverage in the European Union between 1 September 2023 and 15 April 2024 shows a median coverage of only 12.0% among people aged > 60 years and 17.1% for people aged > 80 years, with high variation among countries [15]. Moreover, responsiveness to vaccines may be reduced in elderly individuals, resulting in a lower level of protection compared to younger individuals [16]. Understanding the immune response in elderly individuals is crucial for developing targeted interventions and vaccine strategies to protect this vulnerable population from COVID-19. Although different studies have demonstrated neutralizing activity against BA.2.86 and JN.1 following XBB vaccination [17,18,19], limited information is available regarding T cell responses to these variants [20,21,22], particularly in elderly individuals. In this study, we assessed T cell responses to RBD proteins from the ancestral wild-type, BA.2.86, and JN.1 in a group of fully vaccinated elderly subjects (≥three doses of the COVID-19 mRNA vaccine), who received one booster dose of the updated 2023–2024 COVID-19 vaccine (Pfizer-BioNTech^®^) targeting the XBB.1.5 Omicron variant. This group includes multiply exposed subjects, reflecting the current situation of hybrid SARS-CoV-2 natural and vaccine-induced immunity in a frail population. Simultaneously, we evaluated T cell responses in a group of healthy adults, SARS-CoV-2-unexposed healthcare workers who were vaccinated during the initial 2021 campaign, one month after the second dose of the BNT162b2 mRNA vaccine. This group of COVID-19-naïve individuals represents an interesting control group of healthy subjects with no hybrid immunity to the ancestral SARS-CoV-2 strain.

## 2. Materials and Methods

### 2.1. Study Design and Participants

We enrolled two groups of subjects from two different institutes. The first group (COVID-19-naïve) included 15 healthcare workers from Fondazione IRCCS Policlinico San Matteo, Pavia, Italy, who were previously unexposed to SARS-CoV-2 and vaccinated for the first time with the BNT162b2 vaccine. The first 15 vaccinated healthcare workers who tested negative for SARS-CoV-2 anti-nucleocapsid IgG and who gave consent to participate in this study were consecutively enrolled. In this group, immune responses were examined in peripheral blood mononuclear cells (PBMCs) collected 1 month after the second dose.

The second group (elderly group) included 18 subjects prospectively recruited from 15 January 2024 to 15 March 2024 among elderly subjects admitted to the Medical Department of a northern Italian hospital (ASST Sette Laghi, Varese). Inclusion criteria were: (i) having received 1 booster dose of the updated 2023–2024 COVID-19 vaccine; (ii) aged 65 years or older; (iii) having completed a primary vaccination cycle with ≥3 doses of the COVID-19 mRNA vaccine; and (iv) no SARS-CoV-2 infection as assessed by a COVID-19 rapid antigen test at the time of recruitment and the absence of symptoms compatible with COVID-19. Exclusion criteria were: (i) documented SARS-CoV-2 infection in the last 6 months and (ii) ongoing therapy with glucocorticoids and/or immunosuppressive medications. A diagram showing the selection criteria for the elderly subjects is reported in Supplementary Figure S2. At least 14 days after the booster targeting the XBB.1.5 omicron subvariant, a 10 mL sample of whole blood was obtained from each participant and collected into an EDTA tube for PBMC isolation.

All subjects signed informed consent. The study protocol conformed to the ethical guidelines of the 1975 Declaration of Helsinki and was approved by the Ethics Committee of Fondazione IRCCS Policlinico San Matteo (document number 20210006653, date of approval 13 January 2021) and by the Ethics Committee of Insubria (protocol code 29/2022, date of approval 19 April 2022). The following clinical data were recorded: age, sex, main comorbidities (diabetes, chronic obstructive pulmonary disease [COPD], cardiovascular disease [CVD], chronic renal failure, immunosuppressive conditions such as autoimmune diseases, and neoplasms), primary hospitalization diagnoses, exposure to SARS-CoV-2 infection, and flu and COVID-19 vaccination history. Clinical information was collected from hospital medical records.

### 2.2. Peptide Pools and Proteins

Fifty-three 15-mer peptide pools overlapping by 11 amino acid residues, covering the RBD of the ancestral Wuhan sequence (GenBank: MN_908947) and BA.2.86 were obtained from JPT (EPI_ISL_18096761, region: 319-541). Whole RBD proteins from the ancestral Wuhan, BA.2.86, and JN.1 strains were obtained from Proteogenix (Schiltigheim, France). Supplementary Figure S3 shows spike protein mutations in the three strains.

### 2.3. Ex Vivo Enzyme-Linked Immunospot Assay (ELISpot Assay)

Antigen-specific T cell responses were evaluated by IFN-γ detection after RBD protein or RBD overlapping 15-mer peptide stimulation in an ELISpot assay according to the manufacturer’s instructions (Mabtech). PBMCs were rested overnight in complete medium and seeded at 2.5 × 10^5^ cells/well in 96-well plates pre-coated with anti-IFN-γ (15 μg/mL; clone 1-D1K; Mabtech, Nacka Strand, Sweden). Test wells were supplemented with the RBD 15-mers or the proteins described above. Negative control wells lacked peptides, and an anti-CD3 mAb (1:1000; clone CD3-2, Mabtech) was used as the positive control. Samples were incubated for 24 h at 37 °C. The plates were then washed five times with PBS (Lonza, Basel, Switzerland) and incubated for 2 h at room temperature with biotinylated anti-IFN-γ. After a further five washing steps, a 1:1000 dilution of alkaline phosphatase-conjugated streptavidin (Mabtech, Nacka Strand, Sweden) was added for 1 h at room temperature. The plates were then washed a further five times and developed for 20 min with BCIP/NBT Substrate (Mabtech, Nacka Strand, Sweden). Spots were counted using an automated ELISpot Reader System (Mabtech). Results were given as IFN-spot-forming units (SFU)/10^6^ PBMC after subtracting spots from negative controls. The positive cut-off was set at 10 IFN-γ SFU/10^6^ PBMC based on the mean of background results obtained with the negative control.

### 2.4. Activation-Induced Marker (AIM) Assay

Cryopreserved PBMCs were thawed, resuspended in complete medium (RPMI 1640 containing 10% FBS, 1% L-glutamine, and 1% penicillin/streptomycin), and rested at 2 × 10^6^ cells for 18 h at 37 °C. Then, 8 × 10^5^ PBMCs/well were plated in 96-well U-plates in complete medium and stimulated with 1 μg/mL of RBD proteins or overlapping peptides. An equivalent amount of DMSO was used as a negative control. After 18 h, PBMCs were washed and stained with CD3, CD56, CD4, CD8, CD134 (OX40), CD137 (41BB), and CD69 (BD Biosciences, Milpitas, CA, USA). A LIVE/DEAD^®^ Fixable Near-IR Dead Cell Stain Kit (Thermo Fisher Scientific, Waltham, MA, USA) was used to determine cell viability. After washing, cells were fixed with 2% paraformaldehyde in PBS and acquired with a FACSCelesta (BD Biosciences) flow cytometer.

RBD-specific T cells were identified by activation-induced markers (AIMs), measured as CD134+ CD137+ co-expression in CD4+ and as CD69+CD137+ co-expression in CD8+ T cell subsets (gating strategy shown in Supplementary Figure S4).

### 2.5. Antibody Measurement

In brief, wild-type, BA.2.86, or JN.1 SARS-CoV-2 RBD recombinant proteins (0.5 μg/mL) were coated onto a 96-well microtiter plate. After overnight incubation at 4 °C, the plate was washed 5 times with wash buffer (0.05% Tween-20-PBS). Subsequently, the plate was blocked with PBS 1× BSA 2%Tween-20 0.01% for 1h at RT. Serum samples were stored at ≤−80 °C until use. After thawing, sera were then diluted 1:50 in 2% BSA 0.01% Tween-20 PBS buffer and transferred into test wells. Negative controls were human sera negative for SARS-CoV-2 antibodies. After a 2 h incubation period at 37 °C, plates were washed 5 times to remove unbound components, and anti-human IgG HRP diluted 1:6000 in 2% BSA 0.01% Tween-20 PBS buffer was added. After 1 h of incubation at 37 °C, the plate was washed 5 times with wash buffer. Peroxidase activity was quantified using the substrate 3,3′,5,5′-tetramethylbenzidine (TMB) left for 20 min at RT. The reaction was subsequently blocked with 2N H_2_SO_4_ and optical density was measured at wavelengths of 450 nm and 620 nm using a plate reader (Biochrom EZ Read 400 microplate-reader, Thermo Fisher Scientific Inc., Göteborg, Sweden).

### 2.6. Statistical Analysis

Statistical analysis and graphical presentations were performed using GraphPad Software 10 (GraphPad Software Inc., La Jolla, CA, USA). Statistical differences between data within the same group were assessed by the non-parametric Friedman test followed by Dunn’s multiple comparisons or by the Wilcoxon matched-pairs signed-rank test. The Mann–Whitney U test was used to compare differences between two groups. The Shapiro–Wilk test was used to determine whether the data were normally distributed. The Fisher’s exact test was used to compare frequencies of responders to stimulation above the cut-off threshold (SFU > 10 for the ELISpot assay, median values obtained after stimulation with the vehicle control for the AIM assay, 2 for the Stimulation Index).

## 3. Results

### 3.1. Study Participants

Between January and March 2024, 18 vaccinated elderly patients with a mean age of 81.5 ± 5.5 years were enrolled. Twelve subjects (66.6%) were male. The primary COVID-19 mRNA vaccination cycle was completed with three doses in two subjects, four in thirteen, and five in three, with the last dose at a median of 16 ± 3 months before the XBB.1.5 vaccine; 94.5% of the subjects also received a flu vaccination at the same time as the XBB.1.5 vaccination. Supplementary Figure S5 shows the timeline of SARS-CoV-2 vaccination and blood sampling in the two groups. All subjects reported previous COVID-19 infection with mild symptoms during the last 3 years (at least 1 year before the XBB.1.5 vaccine booster). The median time between the XBB.1.5 vaccine booster and cell collection was 2.8 ± 1.1 months. Seventeen individuals (94.4%) suffered from cardiovascular diseases, four had diabetes (22.2%), three had chronic obstructive pulmonary disease (16.6%), and three had chronic renal failure (16.6%). None were immunosuppressed or under immune therapy with glucocorticoids and/or immunosuppressive medications. Between March and April 2021, 15 healthcare workers with a mean age of 40.8 ± 13.3 were enrolled. Eight (53.3%) were male and were recruited from San Matteo Hospital in Pavia. None were exposed to SARS-CoV-2, as shown by the absence of anti-nucleocapsid IgG (data not shown). They were vaccinated during the initial 2021 campaign with two doses of the mRNA Pfizer-BionTech BNT162b2 vaccine. This group of COVID-19-naïve individuals were immunized with the Wuhan strain and PBMCs were collected 1 month after the second dose.

Table 1 shows the demographic and clinical features of the subjects included in this study.

### 3.2. Anti-RBD IgG Antibodies

**Figure 1:**
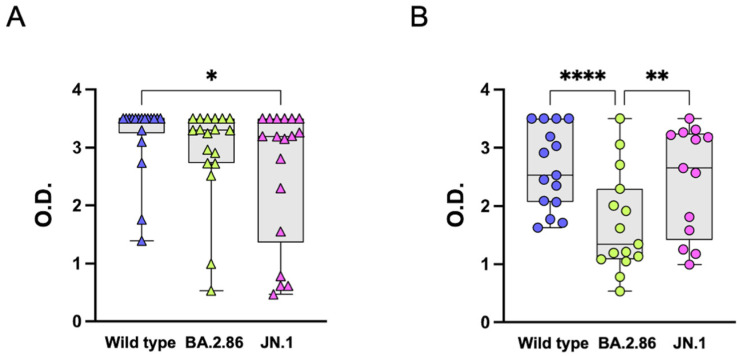
**Anti-RBD IgG responses to ancestral wild-type, BA.2.86, and JN.1 Omicron subvariants.** (**A**) Serum IgG from XBB.1.5-vaccinated elderly subjects (triangles) specific for the RBD ancestral wild-type (blue symbols), BA.2.86 (green symbols), or JN.1 (pink symbols) proteins. (**B**) Serum IgG from BNT162b2 mRNA-vaccinated, COVID-19-naïve healthcare workers (circles) specific for the RBD ancestral wild-type (blue symbols), BA.2.86 (green symbols), or JN.1 (pink symbols) proteins. RBD—receptor-binding domain, O.D.—Optical Density. The one-way Friedman with Dunn’s multiple comparison test was used to compare data. * *p* < 0.05; ** *p* < 0.01; **** *p* < 0.0001.

Compared with BA.2.86, the JN.1 spike protein is characterized by a solitary additional mutation, L455S, located in the RBD. This mutation provides JN.1 with enhanced infectivity, retaining the immune-evasive mutations of BA.2.86. This prompted us to assess the impact of this mutation on T and B cell responses to the RBD in the two groups. To assess the development of RBD-specific IgG antibodies to BA.2.86 and JN.1 variants following SARS-CoV-2 vaccination, we used a qualitative ELISA assay. Serum samples from the two groups of subjects were incubated in wells coated with RBD proteins of the ancestral wild-type, BA.2.86, or JN.1 variants. Comparable IgG reactivities were found in the elderly group for the wild-type strain and the BA.2.86 subvariant, while JN.1 subvariant-specific IgG reactivities were lower compared with the wild-type (Figure 1A). In the COVID-19-naïve group, RBD-specific IgG reactivities were significantly reduced when tested against the BA.2.86 subvariant when compared with the ancestral wild-type strain. Surprisingly, the ancestral strain and the JN.1 omicron subvariant were equally recognized by anti-RBD IgG (Figure 1B).

### 3.3. Reduced T Cell Responses to the BA.2.86 Variant in Elderly Subjects

**Figure 2:**
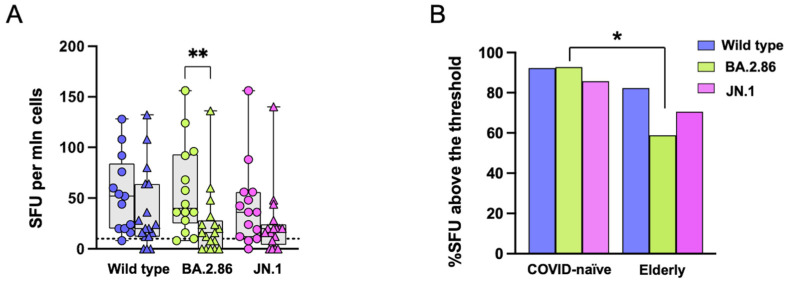
**Interferon (IFN)γ secretion following stimulation with RBD proteins.** Each data point represents the spot count from one study participant. Results are given as IFN-γ spot-forming units (SFU)/10^6^ PBMC from each study participant. (**A**) SFU in the elderly group (triangles) and in the COVID-19-naïve subject group (circles) following ancestral (blue symbols), BA.2.86 (green symbols), or JN.1 (pink symbols) RBD protein stimulation, after subtraction of the unstimulated control. (**B**) Proportion of responders above the SFU threshold. The positive cut-off was set at 10 IFN-γ SFU/10^6^ PBMC. SFU—spot-forming units; RBD—receptor-binding domain. Statistical differences between groups were assessed by the non-parametric Mann–Whitney U test. Fisher’s exact test was used to compare proportions. * *p* < 0.05; ** *p* < 0.01.

IFN-γ released by PBMCs following stimulation with RBD proteins (wild-type, BA.2.86, or JN.1) was detected by ELISpot. There were no differences in immune responses to the three RBD proteins in both the elderly and the COVID-19-naïve groups (Figure 2A). In the elderly group, median T cell responses to the ancestral Wuhan strain, BA.2.86, and JN.1 RBD proteins were 20 (range: 0–132), 16 (range: 0–136), and 20 (range: 0–140) IFN-γ -SFU/10^6^ PBMC, respectively. For the COVID-19-naïve group, median T cell responses were 48 (range: 8–128), 36 (range: 8–156), and 36 (range: 0–156) IFN-γ SFU/10^6^ PBMC, respectively. Notably, the elderly group showed a significantly reduced response to the BA.2.86 subvariant compared with the COVID-19-naïve group, despite the presence of this Omicron subvariant in the new vaccine (Figure 2A). The frequency of subjects responding above the threshold (SFU > 10) is illustrated in Figure 2B.

**Figure 3:**
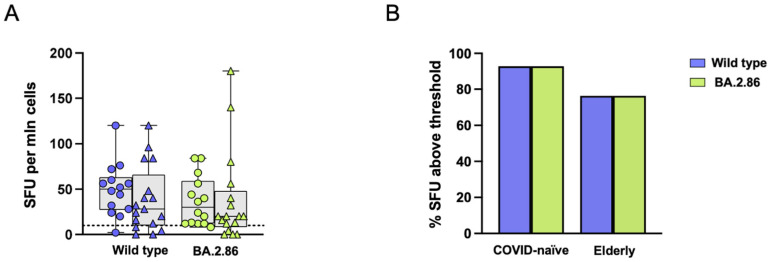
**Interferon (IFN)γ secretion following stimulation with RBD overlapping peptides.** Each data point represents the spot count from one study participant. Results are given as IFN-γ spot-forming units (SFU)/10^6^ PBMC. (**A**) No differences in SFU were observed in the elderly group (triangles) or the COVID-19-naïve group (circles) following ancestral (blue symbols) or BA.2.86 (green symbols) RBD peptide stimulation after subtraction of the unstimulated control. (**B**) The proportion of responders above the threshold was not significantly different following ancestral or BA.2.86 peptide stimulation in the elderly group compared with the COVID-19-naïve group. The positive cut-off was set at 10 IFN-γ SFU/10^6^ PBMC. SFU—spot-forming units; RBD—receptor-binding domain Statistical differences between groups were assessed by the non-parametric Mann–Whitney U test. Statistical differences within the same group were assessed by the Wilcoxon matched-pairs signed-rank test.

Stimulation with overlapping peptides from the RBD of the ancestral strain and the BA.2.86 subvariant induced similar SFU values in both groups. No differences were observed between the two groups in wild-type- and BA.2.86-induced immune responses (Figure 3A). In the elderly group, median T cell responses to ancestral strain and BA.2.86 peptides were 28 (range: 0–120) and 20 (range: 0–180) IFN-γ -SFU/10^6^ PBMC, respectively. Median T cell responses to ancestral strain and BA.2.86 peptides in the COVID-19-naïve group were 50 (range: 2–120) and 30 (range: 8–84) IFN-γ SFU/10^6^ PBMC, respectively. The frequency of subjects responding to stimulation above the threshold (SFU > 10) is illustrated in Figure 3B.

### 3.4. CD4 T Cell Responses Against the JN.1 Protein Are Reduced in the COVID-19-Naïve Group Compared with Other Viral Proteins

**Figure 4:**
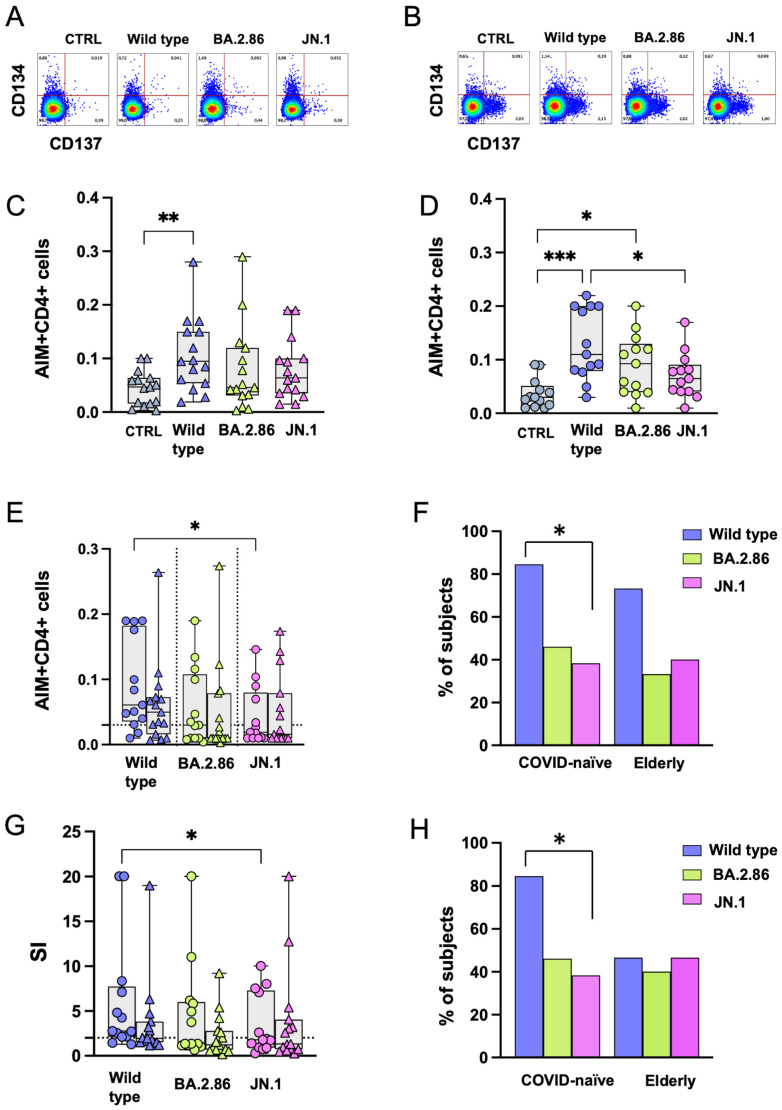
**Expression of Activation-Induced Markers (AIMs) in CD4+ T cells following stimulation with RBD proteins.** Representative AIM dot plots from (**A**) one elderly and (**B**) one COVID-19-naïve subject. Frequency of AIM-expressing CD4+ T cells following ancestral wild-type (blue symbols), BA.2.86 (green symbols), or JN.1 (pink symbols) RBD stimulation in (**C**) the elderly group (triangles) and (**D**) in the COVID-19-naïve group (circles). (**E**) Reactive CD4+ T cells following wild-type (blue symbols), BA.2.86 (green symbols), or JN.1 (pink symbols) RBD protein stimulation in COVID-19-naïve subjects (circles) and elderly subjects (triangles) after subtraction of the negative control. (**F**) Frequency of subjects responding above the threshold in the two groups. (**G**) Stimulation Index in the elderly group and in the COVID-19-naïve group. (**H**) Frequency of subjects responding 2-fold above the stimulation index. RBD—receptor-binding domain; SI—Stimulation Index. The one-way Friedman with Dunn’s multiple comparison test was used to compare data within the same group. Statistical differences between groups were assessed by the non-parametric Mann–Whitney U test. Fisher’s exact test was used to compare proportions. * *p* < 0.05; ** *p* < 0.01; *** *p* < 0.001.

The ELISpot assay does not distinguish which cell type secretes IFN-γ. To identify which cells responded to RBD variants, we assessed activation-induced marker (AIM) expression after stimulation with RBD proteins and RBD overlapping peptides (OPs). CD134 and CD137 co-expression was used to evaluate CD4 RBD-specific T cell responses. In both groups, stimulation with RBD proteins led to a significantly increased AIM frequency in CD4+ T cells following stimulation with the ancestral RBD variant compared with the negative control (Figure 4A–D). Moreover, COVID-19-naïve subjects showed a significantly increased frequency of AIM+ CD4 T cells after BA.2.86 stimulation compared with the negative control (Figure 4D). However, a reduced frequency of AIM+ CD4 T cells was observed after JN.1 stimulation compared to the wild-type strain (Figure 4D), which was confirmed by subtracting the AIM responses in negative control (DMSO) cultures (Figure 4E).

Using a cut-off threshold based on the median values obtained after stimulation with the vehicle control, the proportion of responders above the threshold after JN.1 stimulation was significantly reduced compared with the wild-type RBD in the COVID-19-naïve group, while no differences were observed between the two groups (Figure 4F). In addition, the Stimulation Index (SI), calculated by dividing the percentage of AIM+ cells in stimulated cultures against the percentage of AIM+ cells in the DMSO control, was significantly reduced in the COVID-19-naïve group following JN.1 stimulation compared with the wild-type strain (Figure 4G). The frequency of CD4 T cell responders (SI > 2) was significantly reduced after JN.1 stimulation compared with the ancestral wild-type strain in the COVID-19-naïve group (Figure 4H).

Thus, CD4 T cell responses against the JN.1 variant were reduced in the COVID-19-naïve group, while responses to BA.2.86 and the wild-type proteins remained similar. In contrast, elderly subjects did not show significant CD4 T cell reactivity to current variants, despite omicron XBB.1.5 vaccination.

### 3.5. CD4+ T Cell Responses Against BA.2.86 Are Reduced in the Elderly Group

**Figure 5:**
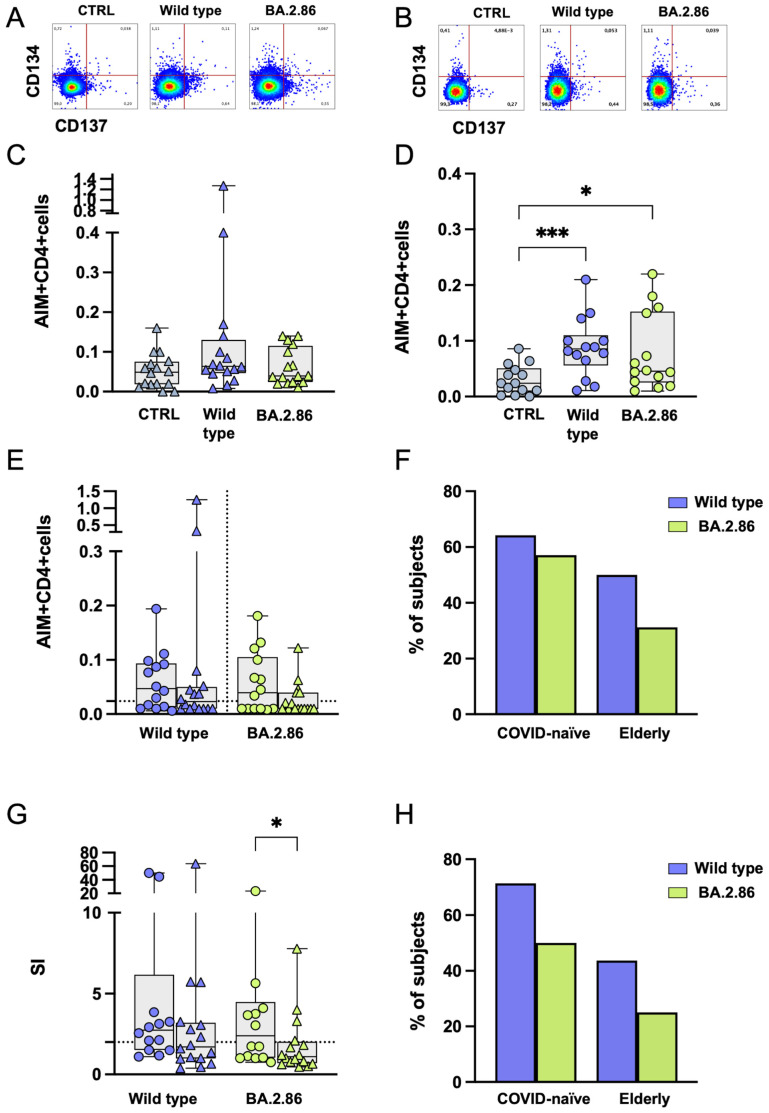
**Expression of Activation-Induced Markers (AIMs) in CD4+ T cells following stimulation with RBD overlapping peptides.** Representative AIM dot plots from (**A**) one elderly and (**B**) one COVID-19-naïve subject. Frequency of AIM-expressing CD4+ T cells following stimulation with ancestral RBD wild-type (blue symbols) or BA.2.86 (green symbols) overlapping peptides in (**C**) the elderly group (triangles) and (**D**) in the COVID-19-naïve group (circles). (**E**) RBD-reactive CD4+ T cells following stimulation with wild-type (blue symbols) or BA.2.86 (green symbols) RBD overlapping peptides in COVID-19-naïve and elderly subjects after subtraction of the negative control. (**F**) Frequency of subjects responding above the threshold in the two groups. (**G**) Stimulation Index in the elderly group and in the COVID-19-naïve group. (**H**) Frequency of subjects responding 2-fold above the stimulation index. RBD—receptor-binding domain; SI—Stimulation Index. Statistical differences between data within the same group were assessed by the non-parametric Friedman test followed by Dunn’s multiple comparisons test. The one-way Friedman with Dunn’s multiple comparison test was used to compare data within the same group. Statistical differences between groups were assessed by the non-parametric Mann–Whitney U test. Fisher’s exact test was used to compare proportions. * *p* < 0.05; *** *p* < 0.001.

OPs were available for the wild-type strain and the BA.2.86 subvariant. Following stimulation with OPs, CD4+ T cell responses were similar for both viral strains, with a significantly higher level of reactive CD4+ T cells compared with the negative control in the COVID-19-naïve group only (Figure 5A–D). No differences were observed in the frequency of CD4+ T cells after subtracting the proportion of those expressing AIMs in negative control (DMSO) cultures (Figure 5E). The proportions of subjects above the threshold after stimulation with RBD proteins are shown in Figure 5F. The elderly group showed a significantly reduced SI after BA.2.86 stimulation compared with the COVID-19-naïve group (Figure 5G). Figure 5H shows the proportion of CD4+ T cell responders (SI > 2) in the two groups following RBD stimulation.

### 3.6. COVID-19 Vaccination Was Unable to Induce an Effective CD8+ T Cell Response Against the Ancestral Wild-Type or Current Variants in SARS-CoV-2-Unexposed and Elderly Individuals

**Figure 6:**
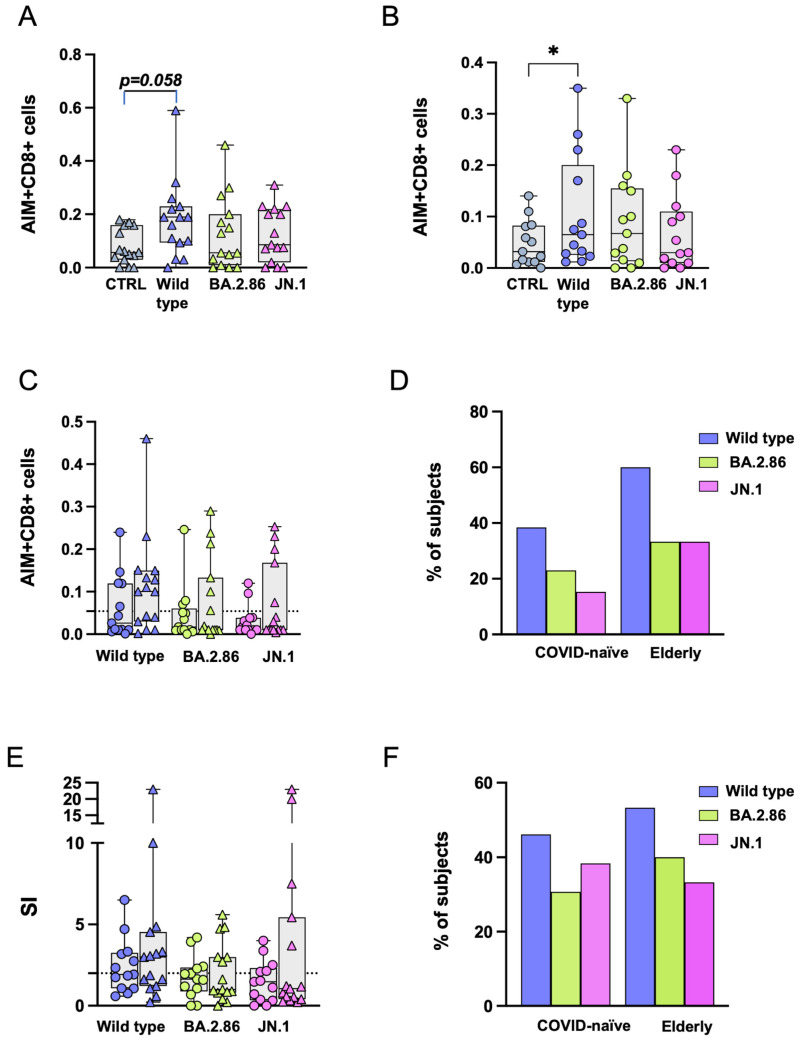
**Expression of Activation-Induced Markers (AIMs) in CD8+ T cells following stimulation with RBD proteins.** Frequency of AIM-expressing CD8+ T cells following ancestral wild-type (blue symbols), BA.2.86 (green symbols), or JN.1 (pink symbols) RBD protein stimulation in (**A**) elderly subjects (triangles) and in (**B**) COVID-19-naïve subjects (circles). (**C**) RBD-induced reactive CD8+ T cells following RBD wild-type (blue symbols), BA.2.86 (green symbols), or JN.1 (pink symbols) protein stimulation in COVID-19-naïve and elderly individuals after subtraction of the negative control. (**D**) Frequency of subjects responding above the threshold in COVID-19-naïve and elderly groups. (**E**) Stimulation Index in the elderly group and in the COVID-19-naïve group. (**F**) Frequency of subjects responding 2-fold above the stimulation index. RBD—receptor-binding domain; SI—Stimulation Index. The one-way Friedman with Dunn’s multiple comparison test was used to compare data within the same group. Statistical differences between groups were assessed by the non-parametric Mann–Whitney U test. Fisher’s exact test was used to compare proportions. * *p* < 0.05.

RBD-specific CD8+ T cell responses were assessed through CD69 and CD137 co-expression analysis. In elderly subjects, no differences were observed in the frequency of AIM-positive CD8+ T cells after stimulation with RBD variants compared with the negative control (Figure 6A), while a significantly increased frequency of AIM-positive CD8+ T cells was observed after stimulation with the ancestral wild-type RBD variant compared with the negative control in the COVID-19-naïve group (Figure 6B).

No differences in antigen-specific AIM responses were observed after subtracting AIM responses in negative control (DMSO) cultures (Figure 6C). Using a cut-off threshold based on the median values obtained after stimulation with the vehicle control, the proportions of subjects above the threshold were not significantly different among variants or between the two groups (Figure 6D). In both groups, the SI was not significantly different following wild-type or BA.2.86 RBD protein stimulation (Figure 6E). Figure 6F shows the proportion of CD8+ T cell responders (SI > 2) in the two groups following RBD stimulation.

**Figure 7:**
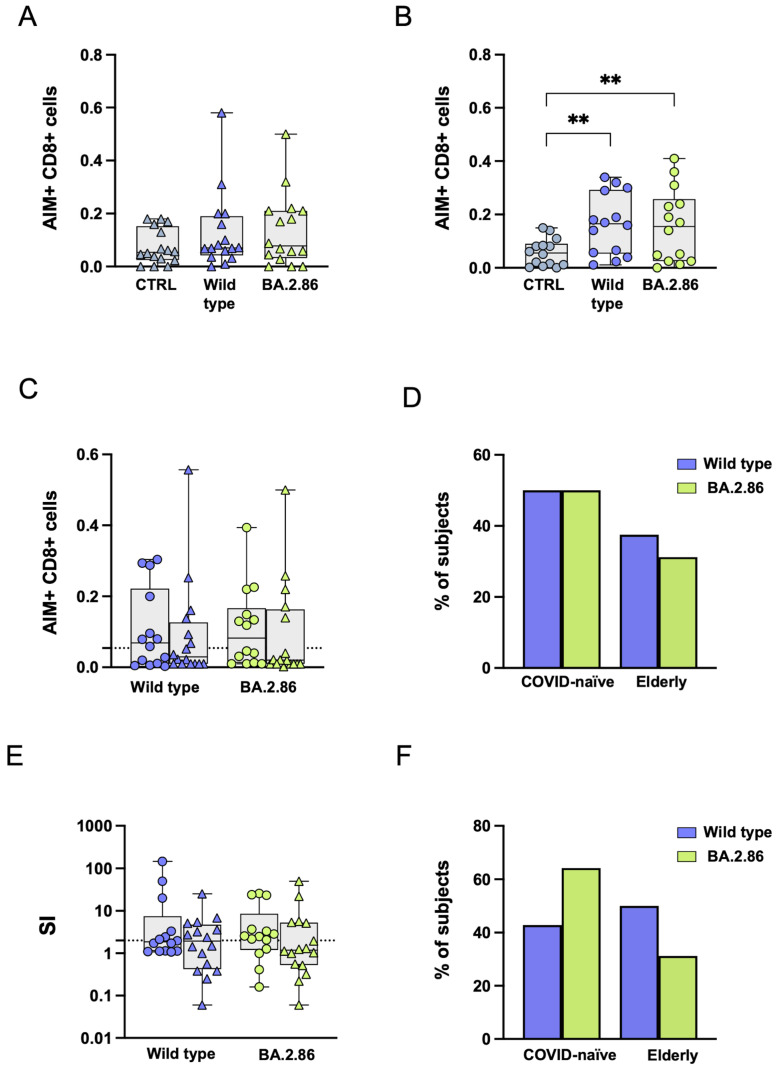
**Expression of Activation-Induced Markers (AIMs) in CD8+ T cells following stimulation with RBD overlapping peptides.** Frequency of AIM-expressing CD8+ T cells following ancestral wild-type (blue symbols) or BA.2.86 (green symbols) RBD protein stimulation in (**A**) elderly subjects (triangles) and in COVID-19-naïve subjects (circles) (**B**). (**C**) RBD-induced reactive CD8+ T cells following stimulation with wild-type (blue symbols) or BA.2.86 (green symbols) RBD overlapping peptides in COVID-19-naïve and elderly subjects after subtraction of the negative control. (**D**) Frequency of subjects responding above the threshold in the COVID-19-naïve and in the elderly groups. (**E**) Stimulation Index in the elderly group and in the COVID-19-naïve group. (**F**) Frequency of subjects responding 2-fold above the stimulation index. RBD—receptor-binding domain; SI—Stimulation Index. The one-way Friedman with Dunn’s multiple comparison test was used to compare data within the same group. Statistical differences between groups were assessed by the non-parametric Mann–Whitney U test. Fisher’s exact test was used to compare proportions. ** *p* < 0.01.

Similarly, stimulation with RBD OPs derived from ancestral wild-type and BA.2.86 variants resulted in a statistically significant increase in responsive CD8+ T cells compared with the negative control only in the COVID-19-naïve group (Figure 7A,B).

No differences were observed after subtracting reactive CD8+ T cell frequencies in negative-control vehicle cultures (Figure 7C), nor in the proportions of responders above the threshold (Figure 7D). The SI showed no significant differences following wild-type or BA.2.86 RBD protein stimulation in either group (Figure 7E). Figure 7F shows the proportion of responders (SI > 2) in the two groups following RBD stimulation.

## 4. Discussion

The immune system tends to weaken with aging, leading to a reduced ability to mount a robust immune response to pathogens, including SARS-CoV-2 [23,24]. Moreover, responsiveness to vaccines may be reduced in elderly individuals, resulting in a lower level of protection compared with younger individuals [25,26]. In this study, we assessed T cell responses to the RBD of ancestral and current Omicron subvariants in a group of elderly subjects who recently received a booster dose containing mRNA encoding for the XBB.1.5 Omicron subvariant. These subjects represent the frail group who developed hybrid immunity to SARS-CoV-2 following natural infection and vaccination. Simultaneously, we also evaluated T cell responses in a group of healthy adults, previously unexposed to SARS-CoV-2, who received two doses of the mRNA Pfizer-BioNTech vaccine during the initial 2021 campaign. Serological analysis revealed that the levels of anti-RBD antibodies in the elderly subject group were equivalent for all three tested variants, indicating a broadly uniform B-cell immune response against each variant. However, despite being vaccinated with the most recent omicron XBB.1.5 mRNA-containing vaccine, elderly subjects did not show increased frequencies of reactive T cells to current variants. Specifically, IFN-γ secretion and AIM SI by CD4+ T cells following BA.2.86 stimulation were reduced in these subjects compared with the COVID-19-naïve group.

Interestingly, an epidemiological study showed reduced XBB.1.5 vaccine-induced immune protection in subjects aged 65 or older infected with BA.2.86 or JN.1 strains. However, the relative risk of hospitalization was not increased compared with other variants [4]. These data should be interpreted with caution in view of the high prevalence of the less aggressive Omicron subvariants in the general population.

The COVID-19-naïve group showed robust CD4+ T cell responses against the BA.2.86 RBD strain. This is in line with a study showing that T cells from subjects with an unknown history of infection or vaccination were able to recognize the BA.2.86 variant [20]. Accordingly, a study from another group observed sustained CD4+ and CD8+ spike-specific T cell responses against SARS-CoV-2 variants, including BA.2.86, in vaccinated healthcare workers. However, unlike our study, the healthcare workers had experienced at least one prior SARS-CoV-2 infection [21]. Moreover, a recent study compared adaptive immune responses to circulating variants (XBB.1.5, EG.5, and BA.2.86) versus previous variants (ancestral, Delta, and BA.5) in subjects with a hybrid SARS-CoV-2 vaccination and infection history. The study showed consistent CD4+ T cell responses across all variants, but a significant reduction in antibody titers, neutralizing antibody levels, and CD8+ T cell responses against circulating variants compared with previous ones [22]. Similarly, our study found that serum anti-RBD IgG from the COVID-19-naïve group showed a reduced ability to recognize BA.2.86 RBD, despite being able to recognize the JN.1 subvariant.

Interestingly, it has been shown that repeated COVID-19 mRNA vaccination causes an increased level of IgG4 spike-specific antibodies, which is associated with the reduced capacity of these antibodies to mediate antibody-dependent cellular phagocytosis and complement deposition [27], and with reduced Ab-mediated natural killer cell activation in older adults [28]. Thus, these findings suggest that IgG4 spike-specific antibodies may play a mechanistic role in dampening the immune response to vaccines in elderly subjects.

Few studies have analyzed T cell responses to the JN.1 subvariant. According to recent findings, T cells from vaccinated individuals are predicted to recognize multiple epitopes on the spike protein of BA.2.86 and JN.1, suggesting that widespread loss of T cell recognition is unlikely [29]. In our study, we observed reduced CD4+ T cell responses against the JN.1 subvariant compared with the ancestral strain, specifically in the COVID-19-naïve group. Notably, when we assessed immune responses using ELISpot assays, no differences were observed in IFN-γ secretion from PBMCs stimulated with ancestral and JN.1 variants in the COVID-19-naïve group, suggesting that other cells may contribute to IFN-γ production, such as natural killer (NK) cells. Indeed, we and others have shown that NK cells exhibit higher effector function upon restimulation with SARS-CoV-2 proteins or peptides in BNT162b2-vaccinated SARS-CoV-2-unexposed individuals [30,31].

Although there were no differences in reactivity between wild-type and JN.1 CD4+ T cells in elderly subjects, it is important to note that only low frequencies of reactive CD4+ T cells were induced by ancestral RBD stimulation in this group. This suggests the existence of broadly reduced immune responses, highlighting the impact of age-related immune senescence. The reduced reactive CD4+ T cell response is indeed clinically relevant because the efficacy of a vaccine depends on the breadth and strength of T cell responses. A reduction in CD4+ T cells has been associated with lower vaccine efficacy, particularly in elderly subjects [32]. Studies have also shown that higher proportions of virus-specific CD4+ T cells and T follicular helper (Tfh) cells are associated with milder COVID-19 symptoms [33], as well as a slower decline in humoral immunity [34]. Moreover, strong CD4+ T cell responses have been associated with better clinical outcomes in COVID-19 patients, while reduced levels of CD4+ T cells correlated with higher rates of breakthrough infections or more severe disease [35].

Another important factor that can explain the lower immune response in the elderly is that mRNA vaccines targeting only one single protein may not provide sufficient protection for older individuals. This is because reliance on a single protein may not account for variants that evade other immune mechanisms, especially against emerging variants or in populations with weaker immune responses. New vaccines using bioengineered attenuated viruses carrying multiple antigens are now under study and may offer stronger immune protection for older adults.

This study has several limitations. First, the sample size is small. This was due to the need to specifically evaluate the contribution of the ancestral vaccine to the recognition of current variants, which required a population of COVID-19-naïve individuals. Second, a control group of younger individuals vaccinated with the XBB.1.5 mRNA-containing vaccine is missing. However, this was due to the fact that in Italy, the XBB.1.5 vaccine campaign was not mandatory, and was primarily recommended for individuals aged 65 and older. As a result, COVID-19 vaccine coverage during 2023–2024 was significantly lower than during the initial vaccine rollout. Additionally, vaccination campaigns for younger subjects are no longer managed by hospital clinics but rather by pharmacies and general doctors, which made recruitment more challenging. Consequently, in the European Union, the median COVID-19 vaccine coverage during 2023–2024 was only 12.0% among people aged over 60 years and 17.1% among people aged over 80 years, with considerable variation among countries [8]. In Italy, at the time of this study, even among patients > 60 years of age, the median vaccination coverage of the XBB vaccine was lower than in the average European population (<10%). This indicates that many individuals remain unprotected from current variants, highlighting the importance of evaluating the ability of the immune system to recognize new variants based on previous vaccines. Despite not being the ideal control group, we think that a group of SARS-CoV-2-unexposed individuals represents a referral group to assess immune responses in SARS-CoV-2-naïve vaccinated persons.

Another limitation is that we did not use overlapping peptides from JN.1, since they were not commercially available at the time of this study. However, whole proteins provide a broader assessment of T cell responses, mimicking natural antigen processing and presentation. This approach captures a wider array of T cell responses, enhancing the understanding of how vaccines or infections elicit hybrid immunity. While overlapping peptides offer precise epitope mapping, whole proteins ensure a comprehensive and natural evaluation of T cell activation, being particularly useful in studies aiming to understand the overall efficacy of vaccines and immune responses to pathogens.

In conclusion, our study shows reduced T cell responses to the BA.2.86 variant in elderly individuals, despite vaccination. These results mirror the age-related decline in immune function. The robust T cell responses in the COVID-19-naïve group to the BA.2.86 variant, comparable to those shown against the ancestral strain, support the idea that initial and booster vaccinations can induce broad and effective T cell-mediated immunity in younger, healthier populations.

CD4+ T cell responses against the JN.1 subvariant were reduced compared with the ancestral strain in COVID-19-naïve individuals. In elderly subjects, reactive CD4+ T cell frequencies were low even with ancestral RBD stimulation, suggesting a generally diminished immune response due to age-related immune senescence.

## Figures and Tables

**Table 1:** Demographic and clinical characteristics of the subjects studied.

Variable	Elderly (*n* = 18)	COVID-19-Naïve (*n* = 15)
Age, years	81.5 ± 5.5	40.8 ± 13.3
Sex, Male	12 (66.6%)	8 (53.3%)
Number of comorbidities	1.6 ± 0.7	None
**Principal cause of hospitalization**		
Trauma	8 (44.5%)	
Heart failure	4 (22.2%)	
Stroke	3 (16.7%	
Intestinal Obstruction	2 (11.1%)	
COPD without infection	1 (5.5%)	
**Comorbidities**		None
CVD	17 (94.4%)	
Diabetes	4 (22.2%)	
COPD	3 (16.6%)	
Chronic renal failure	3 (16.6%)	

Mean ± standard deviation for continuous variables is reported where appropriate; numbers and percentages are used for categorical variables. CVD: cardiovascular disease. COPD: chronic obstructive pulmonary disease.

## Data Availability

The data presented in this study are available upon request from the corresponding author. The data are not publicly available due to privacy restrictions.

## Supplementary Materials

The following supporting information can be downloaded at: https://www.mdpi.com/article/10.3390/vaccines12121451/s1, Figure S1: Timeline of the SARS-CoV-2 outbreaks; Figure S2: Diagram showing selection criteria for the elderly subject group; Figure S3: Spike mutations; Figure S4: Gating strategy; Figure S5: Timeline of vaccination and blood sampling.
