# Supplementary figures and images for: T Cell Responses to BA.2.86 and JN.1 SARS-CoV-2 Variants in Elderly Subjects
Source: Vaccines (Basel). 2024 Dec 23;12(12):1451. doi: 10.3390/vaccines12121451 (PMC11680353; doi:10.3390/vaccines12121451)

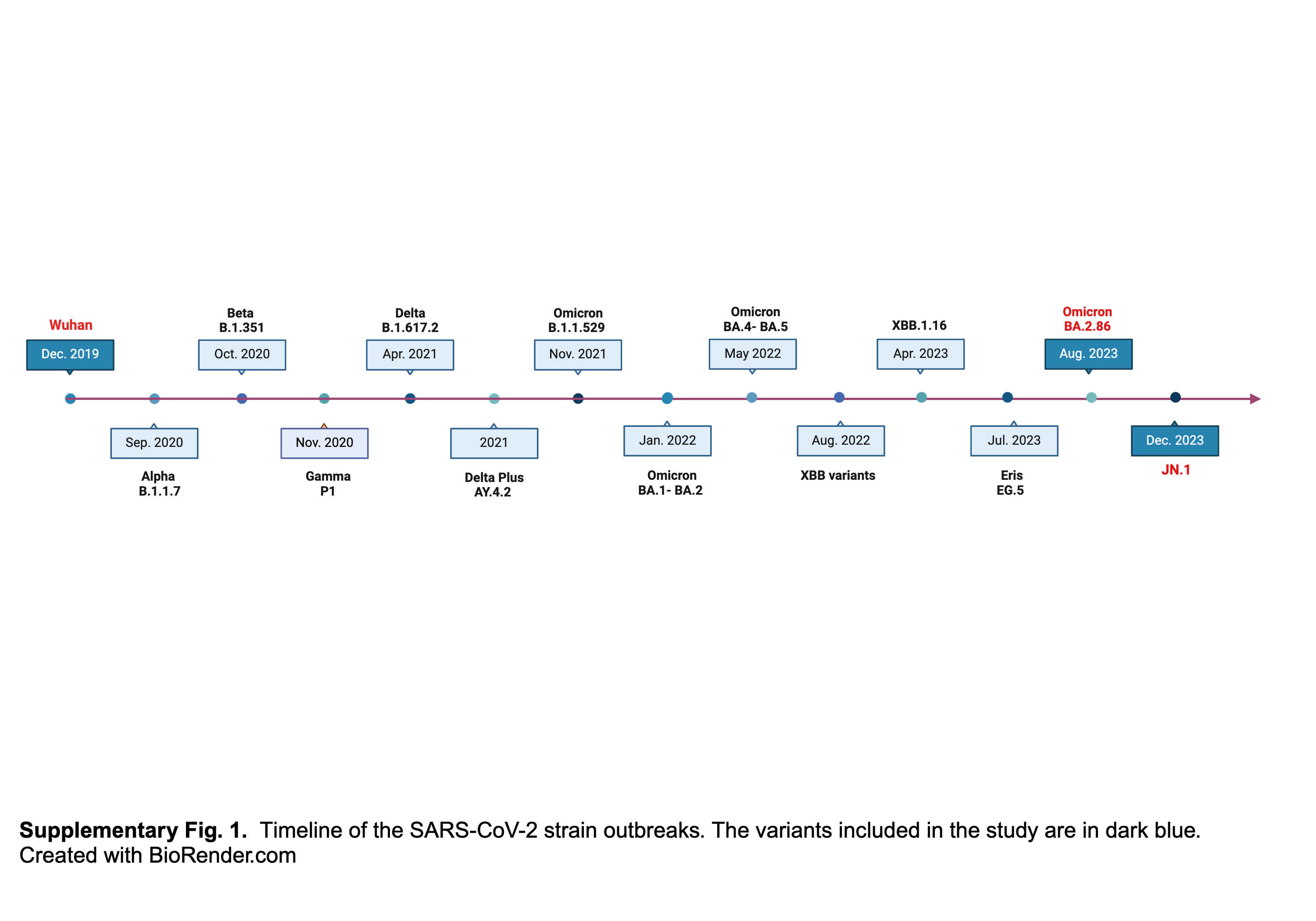

Supplement: Supplementary file 1 [file vaccines-12-01451-s001.zip › Supplementary Fig S1.tiff]

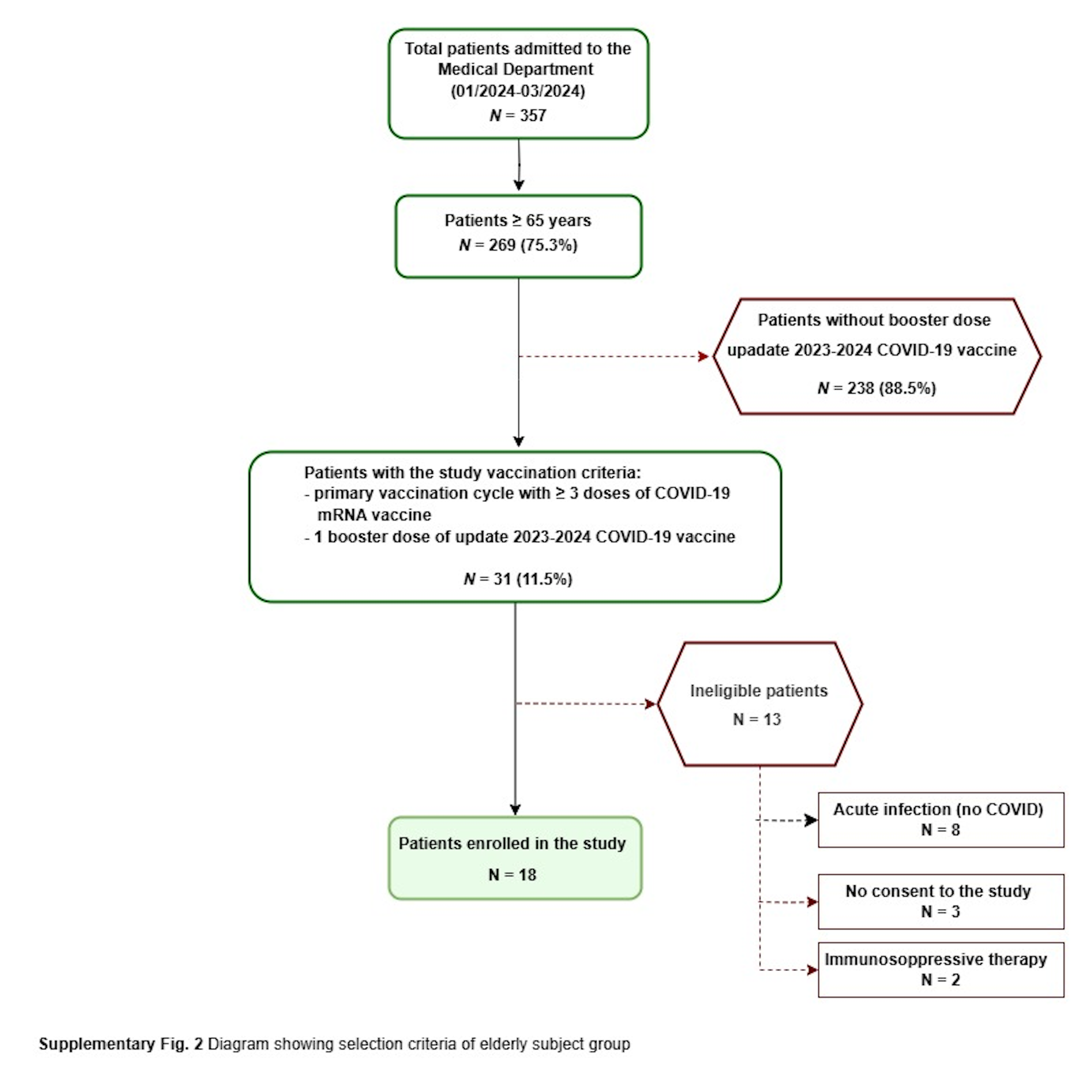

Supplement: Supplementary file 1 [file vaccines-12-01451-s001.zip › Supplementary Fig S2.tiff]

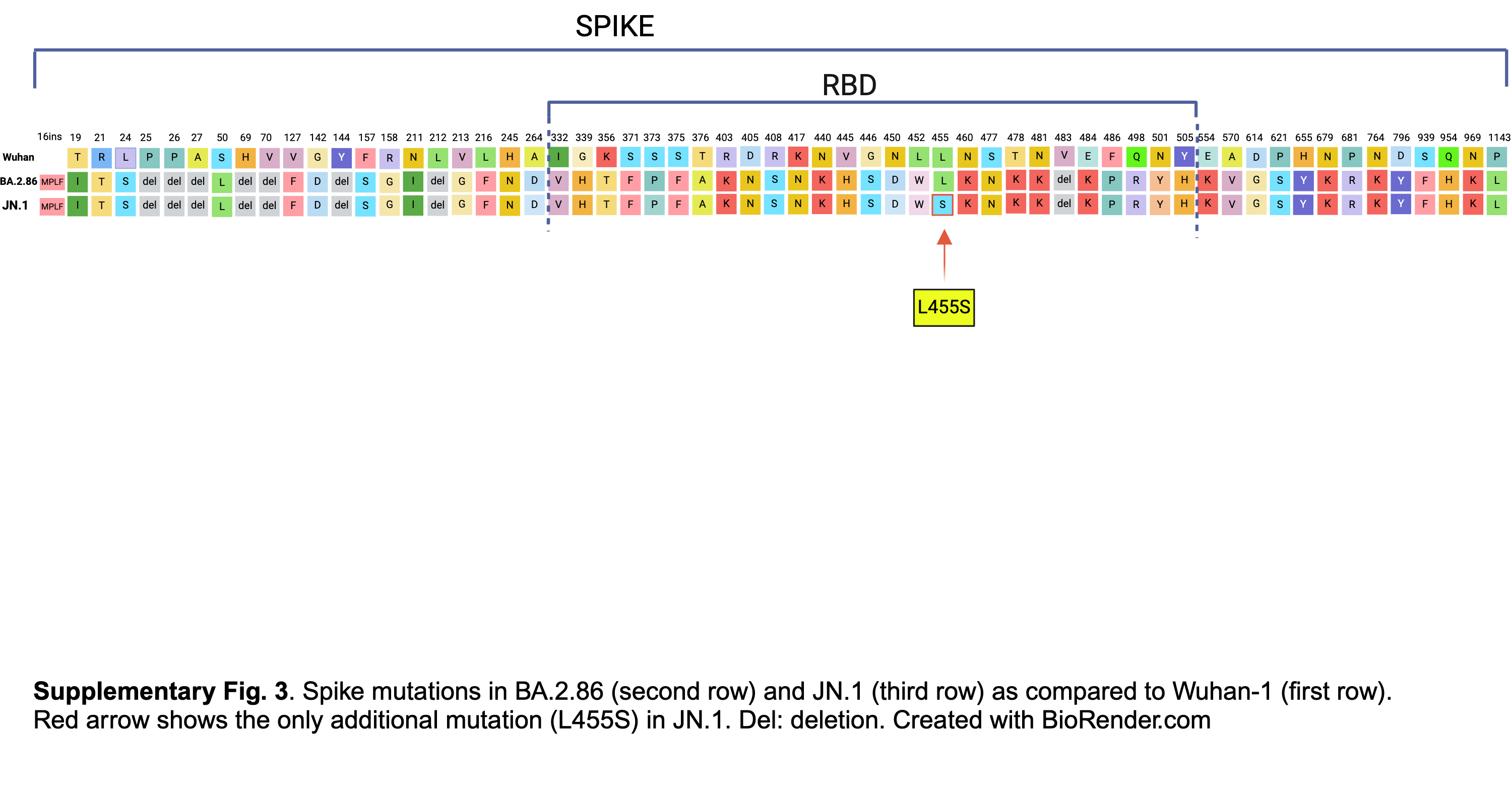

Supplement: Supplementary file 1 [file vaccines-12-01451-s001.zip › Supplementary Fig S3.tiff]

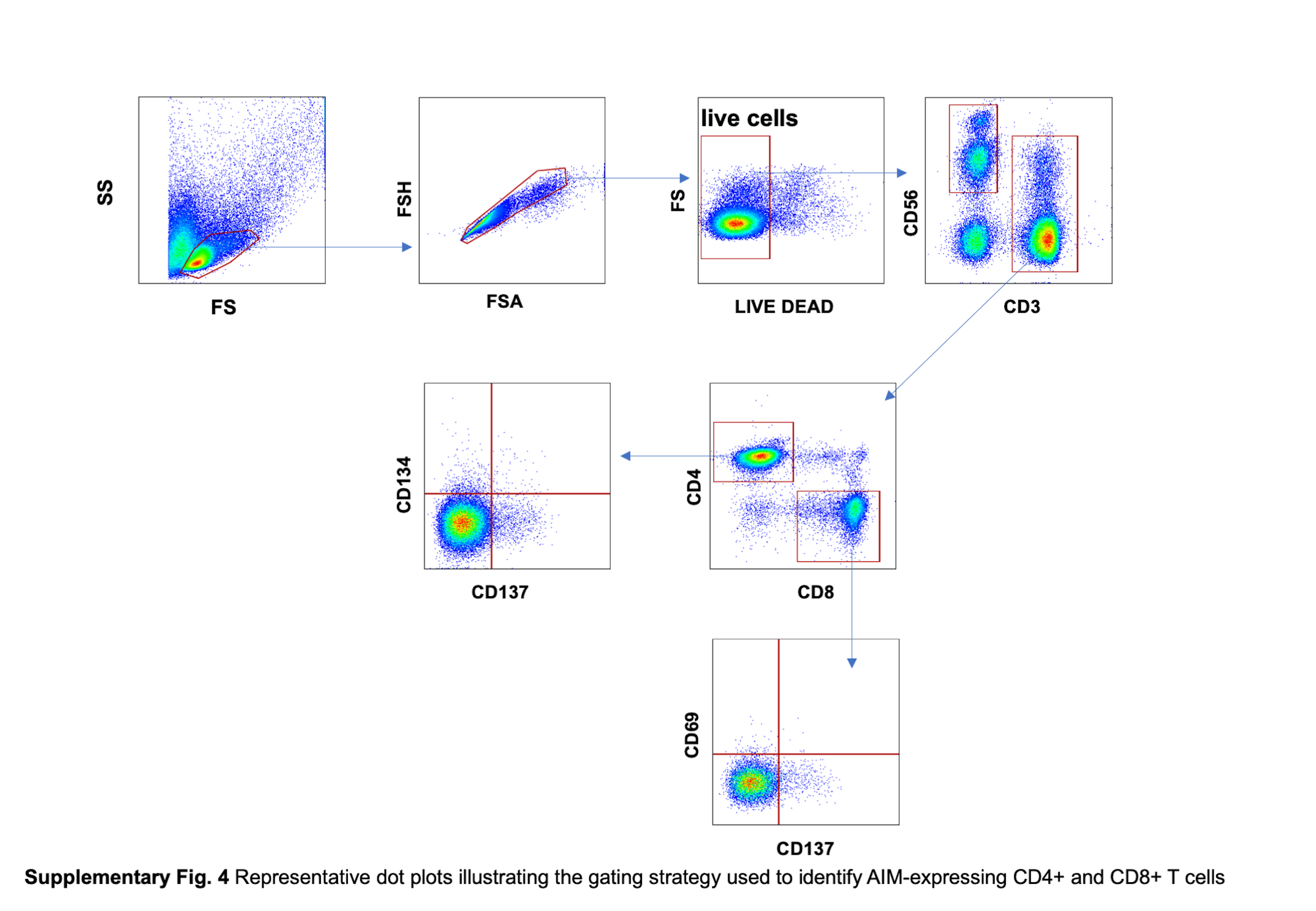

Supplement: Supplementary file 1 [file vaccines-12-01451-s001.zip › Supplementary Fig S4.tiff]

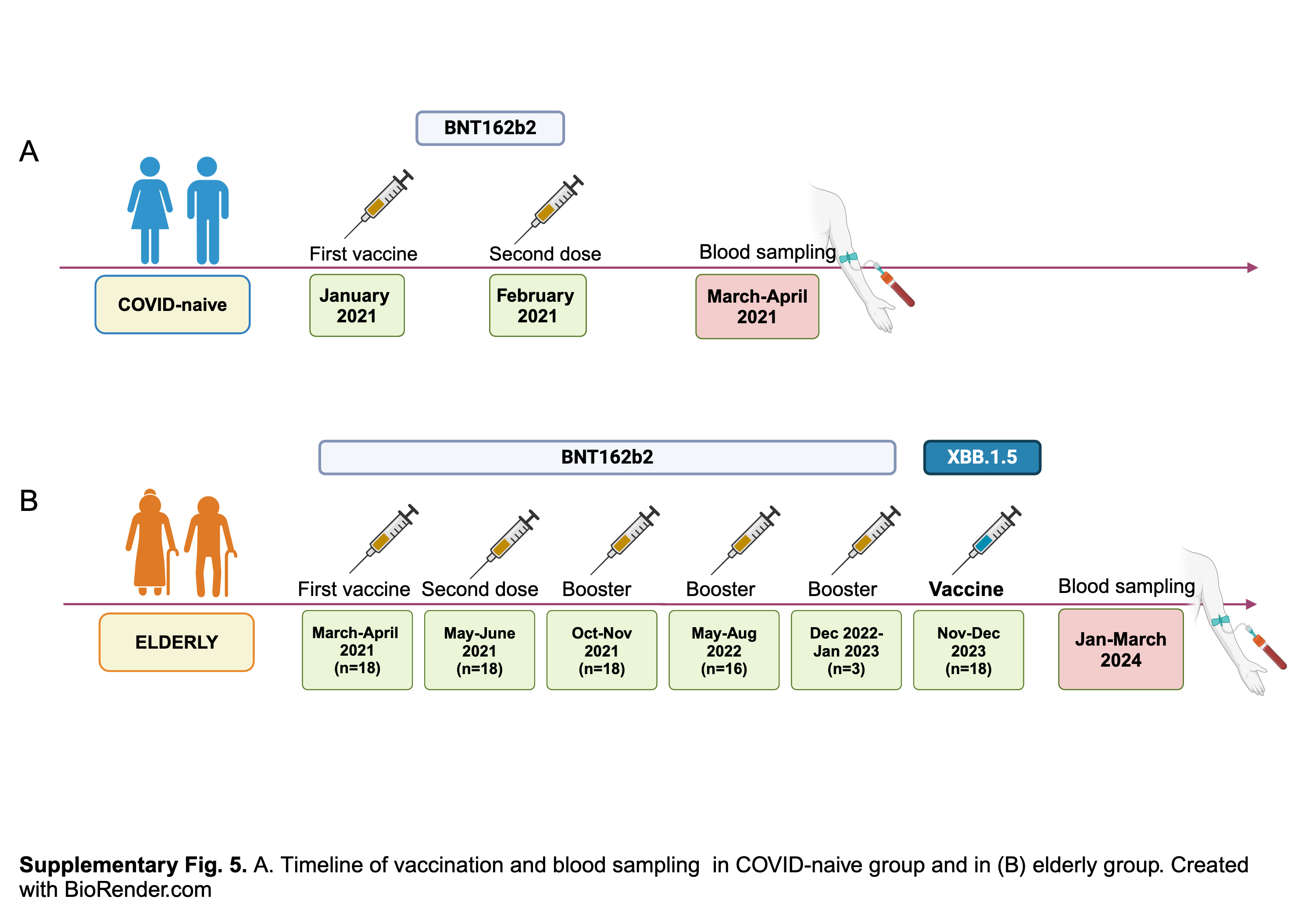

Supplement: Supplementary file 1 [file vaccines-12-01451-s001.zip › Supplementary Fig S5.tiff]
